# Lessons for the clinical nephrologist: lumasiran as the future cornerstone treatment for patients with primary hyperoxaluria type 1?

**DOI:** 10.1007/s40620-022-01435-5

**Published:** 2022-08-20

**Authors:** Valentine Gillion, Karin Dahan, Anaïs Scohy, Arnaud Devresse, Nathalie Godefroid

**Affiliations:** 1 Department of Nephrology, Cliniques Universitaires Saint-Luc, 10, Avenue Hippocrate, 1200 Brussels, Belgium; 2 Department of Genetics, Institut de Génétique et de Pathologie, Gosselies, Belgium; 3 Department of Microbiology, Cliniques Universitaires Saint-Luc, Brussels, Belgium; 4 Department of Pediatric Nephrology, Cliniques Universitaires Saint-Luc, Brussels, Belgium

**Keywords:** Lumasiran, RNA interference therapy, Primary hyperoxaluria type 1

Primary hyperoxaluria type 1 (PH1)—OMIM #259900—is a rare recessive autosomal disorder caused by a deficiency of the liver peroxisomal enzyme alanine-glyoxylate-aminotransferase (AGT), which catalyzes the conversion of glyoxylate to glycine. Reduced AGT activity leads to the conversion of glyoxylate to oxalate (Fig. 1). Oxalate forms insoluble calcium oxalate crystals that accumulate in the kidney and subsequently in other organs, when the kidneys are saturated, leading to systemic oxalosis [1]. The most severe PH1 cases start during the first months of life with rapid development of kidney failure [1]. Before 2020, treatment of PH1 mainly relied on supportive measures including intensive water intake, and on the prescription of vitamin B6 and oral crystallization inhibitors. Despite this, many patients still experience serious and life-threatening complications, especially end-stage kidney disease [1]. To date, PH1 patients with kidney failure can only be cured by dual liver-kidney transplantation with the well-known increased morbidity and mortality risks [2].

**Fig. 1:**
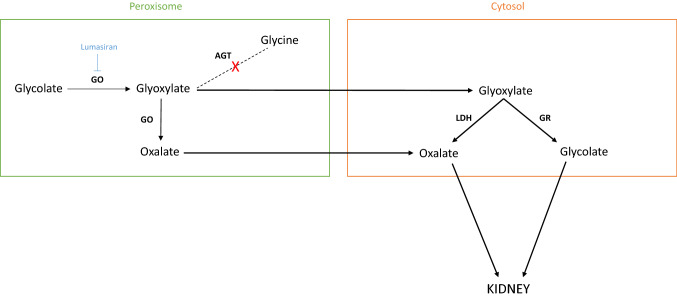
Glyoxylate metabolism in the hepatocytes in primary hyperoxaluria type I. In the peroxisome of normal hepatocytes, glycolate oxidase (GO) catalyzes the conversion of glycolate to glyoxylate. Then alanine-glyoxylate aminotransferase (AGT) catalyzes the conversion of glyoxylate and alanine to glycine and pyruvate and of serine to hydroxypyruvate. In primary hyperoxaluria type 1, glyoxylate accumulates as a result of AGT deficiency and is converted to oxalate by hepatic lactate dehydrogenase (LDH) and GO, and to glycolate by glyoxylate reductase-hydroxypyruvate reductase (GRHPR). Oxalate and glycolate are finally eliminated from the body by the kidneys. Lumasiran inhibits hepatic GO

The following two cases describe the outcomes of two PH1 patients treated with lumasiran, a new innovative therapeutic approach.

## Case 1

**Fig. 2:**
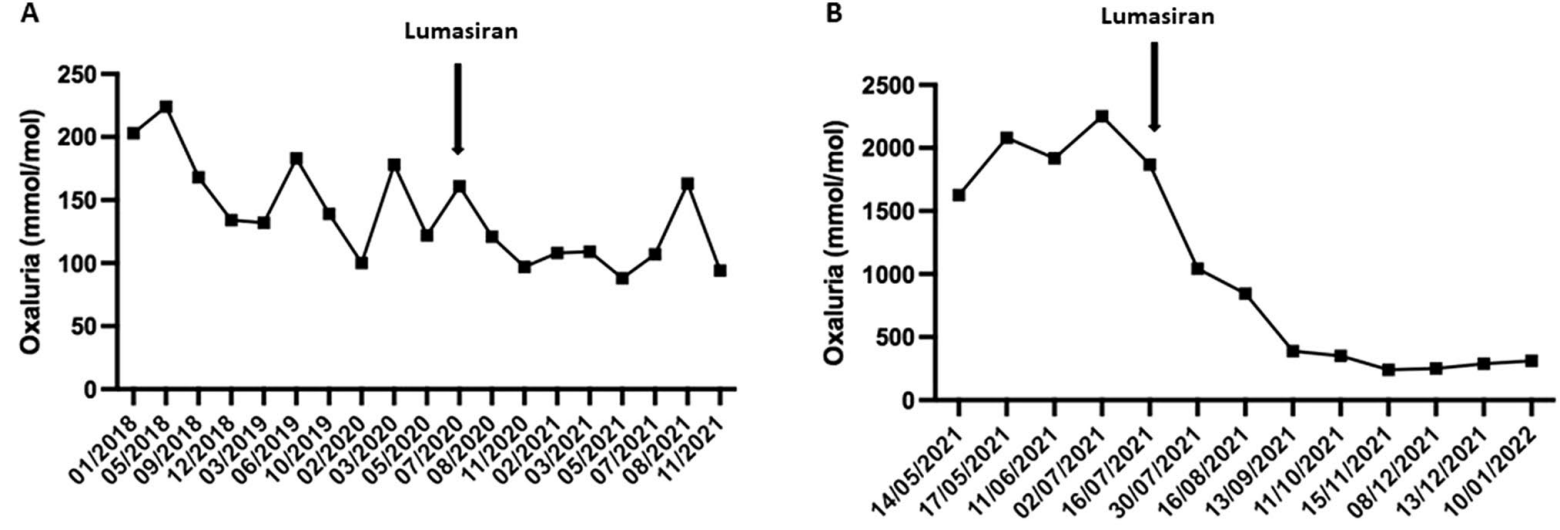
Evolution of urine oxalate levels in patient 1 (**A**) and patient 2 (**B**)

Patient 1, born in October 2012, was fortuitously diagnosed with bilateral nephrocalcinosis just after birth. She had both elevated urinary oxalate/creatinine ratio (Uox/creat) at 896 (normal value for infant < 6 months: 320) mmol/mol (700 mg/g creatinine) and elevated glycolic acid/creatinine ratio at 2744 (normal value < 200) mmol/mol (1829 mg/g). Kidney function was normal. The diagnosis of PH1 was confirmed by genetic testing with compound heterozygous pathogenic variants c.568G > A (p.Pro11Leu) and c.121G > A (p.Gly41Arg) in the *AGXT* gene. A gastrostomy tube was inserted for hyperhydration together with oral treatment consisting of potassium citrate and pyridoxine. In June 2013, nephrocalcinosis was undetectable on ultrasound despite persistent elevated oxaluria at 855 mmol/mol (667 mg/g). In February 2020, kidney ultrasound showed bilateral kidney stones, absent in the previous ultrasound performed two years earlier. In July 2020, she started lumasiran through compassionate use, in addition to the usual conservative measures with hyperhydration, pyridoxine and potassium citrate. Tolerance of lumasiran has been excellent. The mean value of Uox/creat reached 158 (normal value for age < 70) mmol/mol (123 mg/g) between January 2018 and July 2020 (Fig. 2) before starting lumasiran, which decreased thereafter to 108 mmol/mol (84 mg/g) from July 2020 to the present, representing a 31% drop (Fig. 2A). Moreover, lumasiran allowed a reduction in hyperhydration from 3.5 to 2 L/day, from February 2021 onwards; the gastrostomy tube will soon be removed together with discontinuation of the specialized nurse’s visits at school. She is still being treated with lumasiran.

## Case 2

Patient 2, born in March 2021 from consanguineous parents, was diagnosed with PH1 in May 2021 after the discovery of bilateral nephrocalcinosis. The child had elevated UOx/creat at 1626 (normal value for age < 320) mmol/mol (1270 mg/g). PH1 diagnosis was confirmed by a homozygous pathogenic variant (c.33dup) of the *AGXT* gene. Conservative treatment was initiated combining hyperhydration, potassium citrate and pyridoxine. Lumasiran was started through compassionate use in July 2021. Urinary oxalate/creatinine ratios dropped to 1043 mmol/mol (814 mg/g), 844 mmol/mol (659 mg/g), 389 mmol/mol (303 mg/g), and 288 mmol/mol (225 mg/g) at day 15, day 30, month 2, and month 5 after treatment start, respectively (Fig. 2B). Lumasiran tolerance has been excellent and kidney function has remained normal so far. Nephrocalcinosis was stable at month 8. A naso-gastric tube was inserted at treatment initiation to ensure hyperhydration and then removed when urinary oxalate dropped. To date, the patient has shown normal growth and mild developmental delay, with global hypotonia and delayed sitting ability. She is still being treated with lumasiran.

### Lessons for the clinical nephrologist

RNA interference (RNAi) is a natural defense mechanism against invasion of exogenous genes mediated by small interfering RNAs [2]. Synthetic small interference RNA with a specific selected sequence can be designed to pair with endogenous mRNA of a targeted gene leading to its cleavage and subsequent silencing of the encoded protein. RNAi is quite a selective process with a theoretical possibility of off-target effects in which an unrelated gene similar to the target gene can also be silenced [3]. However, there is no clinical evidence of such off-target effects in clinical trials to date [4–7]. Lumasiran is a subcutaneously administered, liver-directed RNA interference therapy targeting the messenger RNA of glycolate oxidase subsequently reducing hepatic oxalate production (Fig. 1). Both safety and efficacy of lumasiran have been assessed in clinical trials (Table 1) [4–7], leading to its approval by the Food and Drug Administration (FDA) and the European Medicines Agency (EMA) in November 2020. Hopefully, lumasiran will improve the current dramatic kidney outcome of PH1 patients, especially if administered early in the course of the disease [1]. In addition, it is likely that this drug will also improve the patient’s quality of life, as illustrated by our cases. Indeed, the first patient had been treated for 8 years with an aggressive conservative therapy including a gastrostomy, and therefore a daily nurse visit at school, and multiple limitations in her everyday life. Thanks to lumasiran started 18 months ago, water intake has been progressively reduced and the gastrostomy will be soon removed. Lumasiran offered the patient a real improvement in her quality of life. In the second patient, the results were even more spectacular in terms of rapid and sustained drop in urinary oxalate levels. The infant currently lives a normal everyday life, has normal kidney function and has experienced no side effects of lumasiran, contrasting with the high rate of early kidney failure described in severe infantile forms. [8]

**Table 1:** Summary of the main results of the Illuminate trials

Trial	Main inclusion criteria	Main findings
Illuminate-A [3, 4]	° Confirmed *AGXT* mutation ° ≥ 6 years ° eGFR ≥ 30 mL/min/1.73 m^2^ Randomized (2:1); 6 months full double-blind, and then 3 months blinded followed by open-label treatment, ***n* = 39**	**Month 6:** 66.9% mean reduction from baseline of 24 h UOx. 13% of patients had improvement of nephrocalcinosis grade **Month 12:** 64.1% mean reduction from baseline of 24 h UOx. 46% of patients had improvement of nephrocalcinosis grade **No major side effects**
Illuminate-B [5]	° Confirmed *AGXT* mutation ° < 6 years ° eGFR ≥ 45 mL/min/1.73 m^2^ in patients aged ≥ 12 months or non-elevated creatinine in patients aged < 12 months ° Open-label single-arm, ***n* = 18**	**Month 6:** 72% mean reduction from baseline of 24 h UOx **No major side effects**
Illuminate-C [6]	° Confirmed *AGXT* mutation ° all ages ° eGFR ≤ 45 mL/min/1.73 m^2^ including patients on chronic dialysis ° Open-label single-arm, ***n* = 21**	**Month 6:** **Cohort A-not on dialysis (*n* = 6):** 33.3% mean reduction from baseline POx Cohort B-on dialysis (*n* = 15): 42.4% mean reduction from baseline POx **No major side effects**

Abbreviations: *eGFR* estimated glomerular filtration rate, *POx* plasma oxalate, *UOx* urine oxalate

### How to use lumasiran

Lumasiran (3 mg/kg) is administered once monthly for the first three months, followed by maintenance doses given once every 3 months beginning 1 month after the last loading dose [4]. Lumasiran is given on top of the conservative measures which include hyperhydration, potassium citrate and vitamin B6 (pyridoxin). The sooner the treatment is initiated, the better the outcome, as documented by Meaux et al. in a small case-series of 3 infants with high oxaluria/creatinine ratios at diagnosis and preserved renal function in 2 of them when lumasiran was initiated [9]. Favorable results were achieved in terms of urinary oxalate level reduction together with good clinical tolerance. The effects of lumasiran on nephrocalcinosis were more mitigated, but this has to be balanced with the short (< 1 year) follow-up of those patients. On the basis of their experience, the authors suggested initiating lumasiran in severe infantile forms as soon as a biochemical diagnosis is strongly suspected, without waiting for genetic confirmation. Similarly, our second patient received lumasiran at a very young age resulting in a dramatic decrease in oxaluria at month 5 but also without any effect on nephrocalcinosis so far, suggesting that nephrocalcinosis improvement may be delayed and that a longer follow-up is required to observe improvement.

### Other innovative treatments in the pipeline.

Nedosiran is an RNA interference agent that inhibits hepatic lactate dehydrogenase (LDH), the enzyme responsible for the common, final step of oxalate production (Fig. 1). Safety, pharmacokinetics, pharmacodynamics, and exposure–response was recently assessed in a phase I study.^Supp^ Stiripentol, an older, safe anti-epileptic drug, also inhibits hepatic LDH, but its efficacy has only been assessed in vitro, in animal models and in one case report.^Supp^ Other strategies, such as the use of *Oxalabacter formigenes* (an anaerobic oxalate-degrading bacteria), chaperone molecules or gene therapy are more or less advanced in clinical development. [2]

### Unresolved questions

The dose for the optimized management of PH1 with lumisaran is currently ill-defined. Of note, in order to achieve a greater reduction in urinary oxalate levels, a transient increase in the administration frequency up to 6 mg/kg/monthly was initiated by Meaux et al. [9] in their patient treated just after birth. In all published cases, lumasiran was given on top of conservative measures (high water intake, potassium citrate with or without pyridoxine). In the present report, oxaluria remains substantially increased in both patients suggesting that additional preventive measures still remain necessary. In this context it is still unknown if lumasiran will allow us to reduce these measures without exerting a negative impact on quality of life of PH1 patients (especially for hyperhydration in infants and children) in long term follow-up. Likewise, it has to be determined whether lumasiran might reduce or delay the use of intensive dialysis regimens that are often required in PH1 patients.

The question which now arises is whether lumasiran could replace liver transplantation within the transplant strategies in PH1 patients with ESKD. In this context, Joher et al. recently published a case of early oxalate nephropathy recurrence after kidney transplantation in a PH1 patient previously treated with lumasiran^Supp.^ A precise algorithm to differentiate patients who need a combined liver-kidney transplantation from those requiring renal transplantation alone is urgently required.

### Main teaching points

Lumasiran is a promising new treatment for PH1, providing great hope for improving the dramatic outcomes of this rare disease. It effectively reduces hepatic oxalate production. We here report encouraging clinical treatment outcomes in one child and one infant followed up in our center. In both cases, Lumasiran was associated with a reduction in urine oxalate levels, improvement in quality of life, with no significant untoward side-effects. Important unresolved questions such as the place for combined liver-kidney transplantation in PH1 patients with ESKD must be addressed in the near future.

## Supplementary Information

Below is the link to the electronic supplementary material.

Supplementary file1 (DOCX 12 kb)
