# Supplementary material for: Lessons for the clinical nephrologist: lumasiran as the future cornerstone treatment for patients with primary hyperoxaluria type 1?
Source: J Nephrol. 2022 Aug 20;36(2):329–33. doi: 10.1007/s40620-022-01435-5 (PMC9998308; doi:10.1007/s40620-022-01435-5)
Supplement: Supplementary file 1 — Supplementary file1 (DOCX 12 kb) [file 40620_2022_1435_MOESM1_ESM.docx]

**Supplementary material (references)**

1. Hoppe B, Koch A, Cochat P, et al. Safety, pharmacodynamics, and exposure-response modeling results from a first-in-human phase 1 study of nedosiran (PHYOX1) in primary hyperoxaluria. *Kidney Int.* 2022;101:626-634
2. Letavernier E, Daudon M. Stiripentol identifies a therapeutic target to reduce oxaluria. Curr Opin Nephrol Hypertens. 2020 Jul;29(4):394-399
3. Joher N, Moktefi A, Grimbert P, Pagot E, Jouan N, El Karoui K, Champy CM, Matignon M, Stehlé T. Early post-transplant recurrence of oxalate nephropathy in a patient with primary hyperoxaluria type 1, despite pretransplant lumasiran therapy. Kidney Int. 2022 Jan;101(1):185-186
